# Concept Analysis of Nursing Surveillance Using a Hybrid Model

**DOI:** 10.3390/healthcare11111613

**Published:** 2023-05-31

**Authors:** Se Young Kim, Mi-Kyoung Cho

**Affiliations:** 1 Department of Nursing, Changwon National University, 20 Changwondaehak-ro, Uichang-gu, Changwon 51140, Republic of Korea; sarakimk@changwon.ac.kr; 2 Department of Nursing Science, College of Medicine, Chungbuk National University, 1 Chungdae-ro, Seowon-gu, Cheongju 28644, Republic of Korea

**Keywords:** surveillance, nursing, concept analysis, hybrid model

## Abstract

This study aims to analyze the concept of nursing surveillance among nurses caring for patients in acute care hospitals in Korea. The conceptual analysis was conducted using the hybrid model proposed by Schwartz-Barcott and Kim. In the theoretical phase, the attributes of nursing surveillance were explored through a literature review. In the fieldwork phase, the attributes of nursing surveillance were derived by analyzing interview materials. In the final analysis phase, nursing surveillance attributes and the related factors were integrated and confirmed. The attributes of nursing surveillance were systematic assessment, pattern recognition, the anticipation of problems, effective communication, decision-making, and performing nursing practice. Based on the theoretical basis of the nursing surveillance concept, this study identified the nursing surveillance concept as perceived by Korean nurses, and examined strategies to promote nursing surveillance.

## 1. Introduction

The Institute of Medicine (IOM) has stated that nurses are the providers most suitable for preventing complications, identifying risks, and responding appropriately to patients [1]. Nursing surveillance is defined as the purposeful and ongoing collection, interpretation, and synthesis of patient data for clinical decision-making in the Nursing Intervention Classification [2]. In acute care hospitals, nursing surveillance is the process of the primary identification of risks to a patient’s health and safety through the purposeful and continuous acquisition, interpretation, and synthesis of patient data for clinical decision-making [3]. Therefore, nursing surveillance is a key intervention in the early detection of adverse events and the prevention of errors [4].

To date, only a few studies have been reported on the concept of nursing surveillance, an emerging research topic in nursing. Kelly and Vincent [3] reviewed the scope of nursing surveillance by categorizing it into a system, target, intervention, and outcome; and Dresser [5] emphasized the importance of nursing surveillance as a nursing intervention to ensure patient safety. Through a systematic literature review, Scotts et al. [6] identified sociotechnical aspects, such as organizational culture and environment, healthcare team communication, and caregiver involvement, that influence nursing surveillance in acute pediatric hospitals based on sociotechnical systems [7], and described the nursing surveillance process as a cyclical process of collecting, interpreting, and synthesizing patient physical, cognitive, and behavioral data; determining risk; and deciding on interventions. Halverson and Tilley [4] described nursing surveillance as a multidimensional concept, and the antecedents of nursing surveillance include a culture of patient safety; nurses’ education, expertise, and staffing; interdisciplinary communication; etc. The consequences of nursing surveillance improved patient safety and reduced complications due to fewer adverse events, reduced nurse burnout, increased satisfaction, reduced experiences of failure to rescue patients, and prevented complications. 

However, there are real differences in the social and technical aspects of nurses’ work in healthcare organizations across countries [8], and the term “surveillance” as a nursing interventions classification (NIC) intervention has not been actively used in clinical practice or nursing education in South Korea, and few studies have examined the surveillance performed by nurses in acute care hospitals. Although surveillance is similar to observation, assessment, and monitoring, there are some differences. In other words, observation is a passive process that does not include the evaluation of collected data, while assessment is collecting and evaluating patient data, but excluding information obtained from family members or other important sources. Monitoring is similar to surveillance, but does not involve the nurse’s cognitive tasks of evaluating, analyzing, and making decisions [9]. On the other hand, Kelly [10] developed and used the NIC’s surveillance activities as a tool to investigate the behavioral attributes of nursing surveillance, but it has been rarely used since then, and the need to develop a nursing surveillance measurement tool that reflects multidimensional attributes of nursing surveillance has been raised [3]. 

Concept analysis is the task of clarifying concepts by identifying appropriate attributes, sorting them into simple factors to clarify the meaning of ambiguous concepts within a theory, and providing precise operational definitions [11]. Schwartz-Barcott and Kim [12] developed the hybrid model of a conceptual analysis method that uses both theoretical and empirical analysis, and it is a useful method for understanding the underlying concepts as a reference index that connects academia and practice, such as nursing learning transfer [11]. As the importance of nursing surveillance is being emphasized for patient safety, it is necessary to study the concept of nursing surveillance in South Korea by analyzing the surveillance interventions performed and perceived by nurses in the field and comparing them with theoretical concepts. To this end, this study aims to analyze the concept of nursing surveillance among nurses caring for patients in acute care hospitals in Korea using the hybrid model of Schwartz-Barcott and Kim [12]. 

## 2. Materials and Methods

The conceptual analysis of nursing surveillance was conducted using the hybrid model proposed by Schwartz-Barcott and Kim [12], which consists of a theoretical phase, a fieldwork phase, and a final analysis phase [11]. In the theoretical phase, we explored the attributes of nursing surveillance through a literature review and identified a tentative definition. In the fieldwork phase, we reconfirmed the attributes of nursing surveillance that appeared in the nursing field through interviews with participants and derived new attributes. In the final analysis phase, the results of the theoretical and fieldwork phases were compared and analyzed, and the nursing surveillance attributes and related factors were integrated and confirmed.

### 2.1. Theoretical Phase

A scoping review was conducted to explore the attributes and definitions of nursing surveillance. The search was conducted from 25 October 2022 to 28 January 2023 across four databases: PubMed, CINAHL, ScienceDirect, and the Korean Studies Information Service System (KISS). Since Halverson and Tilley [4] conducted a systematic review of papers published from 2010 to 2019, the selection criteria in this study were (1) papers published online/offline from 1 January 2020 to 31 December 2022, (2) the language being limited to English and Korean, (3) and the participants of the study being clinical nurses working in an acute hospital setting. The exclusion criteria were papers about nurses working in public health, school health, nursing home, or home care settings. The search terms “nursing surveillance”, “nursing” and “surveillance”, and “nurse” and “surveillance” were used to retrieve the titles and abstracts of articles. No Korean articles were found as a result of the search, but a total of 829 articles in English from PubMed, 183 from CINAHL, and 21 from ScienceDirect were retrieved. After removing duplicates, 19 articles from PubMed, 21 from CINAHL, and 8 from ScienceDirect were selected as nursing-surveillance-related articles. Duplicate articles were excluded based on their titles, and the abstracts of relevant articles to nursing surveillance were reviewed. In alignment with the aim of exploring the attributes of nursing surveillance, this study was not assessed for the quality of the included papers. A total of 10 articles related to the concept of nursing surveillance were selected by reviewing the full-text articles, and were used for the analysis of the theoretical phase of the analysis. 

### 2.2. Fieldwork Phase

To validate the concept and content of nursing surveillance extracted from the literature, the attributes, antecedents, and consequences of the nursing surveillance concept identified in the theoretical phase were checked to see how they manifest themselves in nursing practice. In the fieldwork phase, 10 nurses with more than 5 years of experience with nursing practice at the bedside, who are most knowledgeable and best able to talk about nursing surveillance concepts, were interviewed about their experiences related to nursing surveillance. From 6 January 2023 to 15 February 2023, two researchers (S.Y.K. and M.-K.C) made a meeting appointment with the participants in advance and conducted each interview. Interviewees were convenience-sampled, and an interview schedule was set up with the interviewee to meet in a quiet, undisturbed area at a time when they could focus on the interview after working hours. After an explanation regarding the protection of the interviewee, the study, and that the interview would be recorded, consent was obtained and the interview was conducted. The interview language was conducted in Korean because all the nurses were Korean, and the interview time per interviewee took 1–1.5 h. The interviews included open-ended discussions about the occurrence, antecedents, outcomes, and attributes of nursing surveillance, including the general characteristics of the participants, and were conducted until the data were saturated. The recorded interviews were transcribed verbatim in the participants’ language, and the transcriptions were checked with each participant to ensure their validity.

### 2.3. Final Analysis Phase

The data of the theoretical phase and the fieldwork phase were shared by two researchers, and each researcher independently integrated and organized the definition, antecedents, attributes, and consequences of nursing surveillance in the data of each phase into a shared form. By comparing and analyzing the results of the attributes, antecedents, and consequences of the nursing surveillance concept derived from the theoretical and fieldwork phases, researchers integrated and confirmed the definition, attributes, and dimensions of the concept for nursing surveillance through several meetings to reach a final agreement.

## 3. Results 

### 3.1. Theoretical Phase

#### 3.1.1. The Definition of Surveillance

According to the National Institute of the Korean Language’s Standard Korean Dictionary, “surveillance” is described as “to watch carefully to crack down”, and “watch” is described as “to look down from a high place” [13]. The English word “surveillance” comes from the French word for “watch over” and was originally a military concept [14], but has since been applied to the concept of disease surveillance, which emphasizes the prevention of infectious diseases [3,4].

#### 3.1.2. Definitions of Surveillance in Other Disciplines

Infectious disease surveillance can be defined as a process of systematically and continuously collecting, analyzing, and interpreting data on infectious disease outbreaks and their carriers, and distributing the results to those who need them promptly for use in infectious disease prevention and management. In 2014, the Center for Disease Control and Prevention (CDC) in the United States launched the National Notifiable Diseases Surveillance System (NNDSS) based on information and communication technologies [15]. More recently, the concept of surveillance has focused on individuals, allowing healthcare consumers and providers to engage in surveillance by accessing real-time data, such as heart rate or blood sugar levels, and allowing smartwatches to collect and transmit patient data, such as blood pressure and heart rate, with healthcare professionals responsible for monitoring changes in vital signs [3,4].

#### 3.1.3. The Concept of Surveillance in Nursing

While the goal of disease surveillance is to prevent the spread of disease, the goal of nursing surveillance is to anticipate and prevent adverse patient events. In the acute care setting, nurses must be competent enough to evaluate overwhelming amounts of patient data for clinical decision-making and respond to subtle changes in patient status [4]. Nursing surveillance is the process of purposefully and continuously collecting, interpreting, and synthesizing patient data for clinical decision-making in the acute care setting, with the primary goal of identifying threats to the patient’s health and safety [3]. In the NIC system, surveillance interventions are defined as activities that purposefully and continuously collect, interpret, and synthesize patient data for clinical decision-making [2]. 

#### 3.1.4. Attributes, Antecedents, and Consequences of Nursing Surveillance

**Figure 1:**
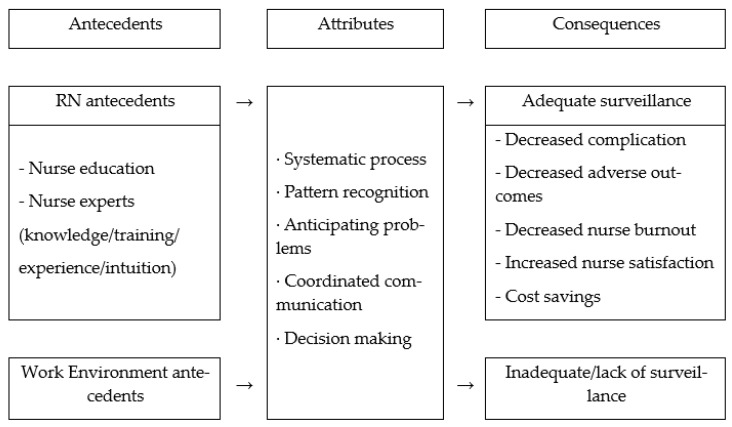
Framework for assessing surveillance.

Kelly and Vincent [3] utilized Rodgers’ [16] evolutionary concept analysis method to analyze the concept of nursing surveillance in the acute care setting based on 18 journal articles and 4 books, focusing on surveillance interventions in the NIC and Omaha from 1985 to 2009. Later, Halverson and Tilley [4] synthesized the antecedents, attributes, and consequences of nursing surveillance by analyzing 24 academic articles on nursing surveillance from 2010 to 2019 using Walker and Avant’s concept analysis method [17], and the results are shown in Figure 1.

Based on 10 journal articles related to nursing surveillance practiced by nurses in acute care settings published since 2020, this study presents antecedents of nurses, workplace aspects, attributes of the concept, factors that promote and hinder nursing surveillance, and the desirable and undesirable consequences of nursing surveillance (Table 1).

In the theoretical phase, the attributes of nursing surveillance include assessment, documentation, decision-making, intervening, communication, continuous process of acquisition, interpretation, recognition, and management of clinical deterioration. The synthesis of physical, behavioral, and cognitive patient data to determine interventions and identify threats to the patient’s health and safety during the course of nursing care [6] include systematic processes, pattern recognition, the anticipation of the problem of instability, coordinated communication [4,23], quality of advice, good working relationships with patients [20], vital sign assessment, chart-reviewing, rounding [23], direct and continuous observation and monitoring, recognizing signs, and accurately assessing probability [25].

### 3.2. Fieldwork Phase

The 10 subjects interviewed during the fieldwork phase were all female, with an average age of 35.60 ± 7.53 years (range: 27–49); 5 had a bachelor’s degree and 5 had a master’s degree, and the positions were 1 head nurse, 3 charge nurses, and 6 general nurses. Their total work experience averaged 13.20 ± 8.03 years (range: 4.92–28.83) and their work departments varied, but the average number of years of experience in the department was 6.48 ± 4.03 years (range: 1.25–15.00), and the confidence on clinical nursing performance was an average of 8.10 ± 1.20 (range: 6–10) (Table 2).

In the fieldwork phase, data collected through the interviews were analyzed using the content analysis method, and the common parts of the subjects’ statements were extracted. Since new themes about nursing surveillance were not found in the interview data of 10 subjects, the data were saturated. The situations that led to nursing surveillance, antecedents from the perspective of nurses and their work environment, attributes of the nursing surveillance concept, and a summary of the subject’s statements according to the attributes of the concept were presented along with the desirable and undesirable consequences of nursing surveillance (Table 3).

#### Attributes of Nursing Surveillance

In the fieldwork phase, the attributes of nursing surveillance were derived as systematic assessment, pattern recognition, anticipation of problems, effective communication, decision-making, and performance of nursing practices.

Systematic assessment includes carefully listening to handover details, the direct confirmation of handover details (vital signs, hemodynamic monitoring, consciousness, appearance, existing lines and drains, catheters, medication, order, lab, video, assessment through open questions to patients and guardians, disease characteristics focus assessment, treatment, and postintervention effect assessment), acquisition of problematic cues, checking of alarms, past events, patient monitoring settings 24 h before handover, and trend of patient condition change, continuous monitoring of the patient condition, planned rounds, and checking the effectiveness of nursing intervention and treatment. The following statement is a good example of a systematic assessment.

“*In any case, I receive a general report on the patient, but when I look at them, it is like this. I mean, if a patient is in critical condition, they have a monitor anyway. Then I look at figures like EKG or SPO_2_ readings on the monitor. I understand that those monitors aren’t always accurate. So, I look at the patient with my own eyes and confirm the patient’s condition. First of all, if the patient’s mental status is not alert, I check their mental status first, and then if their vital signs are somewhat unstable I bring that. If they have a fever, I measure their temperature or take their blood pressure and monitor them if their blood pressure is somewhat unstable. Then intubation, any tubes I have… I check for any incidents or events that the patients may have had. I check the L-tube or Foley catheter and see if it’s in a good immobilization position and then I ask their guardians if there were any events or incidents when the patient was not being monitored and if there is any discomfort. That is how I do it.*”

(RN 3)

Pattern recognition includes arranging handover details, organizing patient assessment data, integrating handover details and assessment data, interpreting the meaning of assessment data, identifying patterns by checking and comparing past and current assessment data, thinking about and finding factors that cause changes in patient condition, recognition of patient problems to be solved during duty, treatment, nursing goals, recalling past patient experiences, and the evaluation of treatment and intervention effects. A good example of pattern recognition is the following statement.

“*Usually the blood pressure was 120–130 and dropped to 100, and the heart rate wasn’t that much higher than the vital signs before, but the blood pressure just dropped slightly. When I asked the patient if they were feeling dizzy, they said they were slightly dizzy at that point. And they said they felt nauseous, so I thought it was bleeding. That was what I was thinking.*”

(RN 1)

The anticipation of problems includes the prediction of the patient’s problem using past and present data, including baseline data, the prioritization of problems to be solved during duty, detection of problems and their prediction by comprehensively judging the assessment data before the patient’s condition changes, the progress of the disease, treatment effects, comparison with past patterns, predicting disease progression and doctor’s orders, preparing patients, and nursing care. The anticipation of problems is best exemplified by the following statement.

“*A patient underwent percutaneous coronary intervention and all of a sudden they complained of chest tightness. I measured vital signs… I noticed signs of an impending arrest. There were signs of it… So, I called for an electrocardiogram… and asked for immediate assistance… I had a feeling that something serious was going to happen. So, I prepared for intubation and put the patient on oxygen. I informed the attending physician that they needed to come quickly…*”

(RN 2)

Effective communication includes communicating with patients and guardians, with handover nurses and duty nurses on the same shift, and notifying doctors of perceived problems, patient conditions, and changes after the intervention. The following statements demonstrate effective communication.

“*A patient had an endoscopy and although they didn’t have any pain, they felt nauseous. As soon as I received a general report, I immediately went to the patient, checked their vital signs, and assessed their symptoms and whether they felt dizzy… Then, I notified the attending physician that they might need to take a look at the X-ray…*”

(RN 1)

“*The patient had a BST of 65 and a BP of around 190… So, the BP was high, I notified the attending physician… When I asked their guardian if this had happened before… the attending physician came and explained the situation… suggested that we consult with the NP and see if there is anything we can do…*”

(RN 2)

“*Doctors are… If there is anything abnormal with the prescription… we find it and report it… A lot of times, doctors don’t even check the results…when I report the results, they’ll order a blood transfusion or something like that… Caregivers or patients talk to the nurse first if they are experiencing discomfort. When the doctor comes rounding to see patients, they don’t say anything, and then if something comes up, they talk to the nurse first…*”

(RN 6)

Decision-making includes the discussion and proposal of the patient treatment plan, participation in patient treatment decision-making, and suggestion of consulting with other departments.

“*First of all, I look at the patient’s initial assessment history…if there’s a record of them being admitted to our hospital, I go through it all, so I do a search, I look at it all… If there’s something that needs to be done, I can notify the attending physician in advance or check it before the patient is admitted, for example, I check whether the levels of CBC, hemoglobin, or creatine level are low or not… The request is made to follow up with the patient again and then ask the patient’s thoughts on it. If necessary, medication will be prescribed.*”

(RN 7)

Nursing practice includes carrying out nursing care according to orders or protocols, educating and preparing patients and guardians about predicted problems, symptom reduction, problem-solving nursing, preparing for and carrying out predicted doctor’s orders, and performing interventions that have to be done on time. A statement that highlights nursing practice well is as follows:

“*I try to do what I can first, for example, if a patient’s saturation drops or they have difficulty breathing, I tell them to take deep breaths and check if the sedative is properly administered. I also check the mucus and make sure the monitoring is done properly. If the patient continues to experience problems, I take measures, such as performing lab tests or changing the ventilator or medication. I am doing it in that order.*”

(RN 4)

“*If a diabetic patient’s guardian says they are unconscious, I first check if they have eaten anything if they have taken insulin before a meal, and if they haven’t, I quickly check their BST and give them 50% glucose.*”

(RN 3)

### 3.3. Final Analysis Phase

In the final analysis phase, the antecedents, attributes, and consequences of nursing surveillance identified in the theoretical and fieldwork phases were compared or analyzed, rearranged, and integrated (Table 4). Systematic assessment includes purposeful and collaborative assessment, acquisition of problematic cues, continuous monitoring of the patient’s condition, planned rounds, and checking the effectiveness of nursing intervention and treatment. Pattern recognition includes organizing and synthesizing handover details and assessment data, interpreting the meaning of assessment data, identifying patterns by checking and comparing past and current assessment data, recognizing patient problems to be solved during duty, and evaluating treatment and intervention effects.

The anticipation of problems includes the prioritization of problems to be solved during duty, the detection of problems and their prediction by comprehensively judging the assessment data before the patient’s condition changes, determining disease progression and treatment effects, comparing current data with past patterns, predicting disease progression and doctor’s orders, preparing patients, and nursing care. Effective communication includes effective and coordinated communication with patients, guardians, handover nurses, duty nurses on the same shift, and other departments, and notifying doctors of perceived problems, patient conditions, and changes after the intervention. Decision-making includes participation in the decision-making aspect of patient treatment plans and exploring alternative solutions. Performing nursing practice includes providing nursing care according to orders, protocols, guidelines, and standards for clinical deterioration and problem-solving, preparing for and carrying out predicted doctor’s orders, and performing interventions that have to be carried out on time.

## 4. Discussion

Nursing surveillance is essential for patient safety [1], and it is necessary to confirm the concept of nursing surveillance to improve it in nursing practice. This study aims to analyze the concept of nursing surveillance using the hybrid model of Schwartz-Barcott and Kim [12]. The attributes of nursing surveillance in this study were identified as “systematic assessment”, “pattern recognition”, “anticipation of problems”, “effective communication”, “decision-making”, and “performing nursing practice.” These results were partially similar to Halverson and Tilley’s [4] nursing surveillance attributes of “systematic process”, “pattern recognition”, “problem anticipation”, “coordinated communication”, and “decision making.” Although there are differences in the social and technical division of nursing work due to historical, economic, and political differences between countries, the work of nurses showed similar characteristics, as seen in the results of Leal and Melo [8].

Additionally, nursing surveillance is a part of the nursing process that involves a variety of activities. Specifically, surveillance involves the evaluation of monitoring parameters, along with the acquisition, integration, and interpretation of data from diverse sources. These sources encompass not only family members and other healthcare team members, but also medical databases and clinical decision support systems [5,26]. In this study, it was confirmed that the attributes of nursing surveillance consist of behavioral factors, such as systematic assessment, effective communication, and performing nursing practice, and cognitive factors, such as pattern, anticipation of problems, and decision-making. As described above, the nursing surveillance can be confirmed as a comprehensive nursing intervention.

In this study, “systematic assessment” was similar to Halverson and Tilley’s [4] “systematic process”, in which nurses performed continuous and repetitive tasks, such as handover, vital sign measurement, direct patient observation, verification of test results, focusing on assessment, questioning patients and guardians, reviewing medical histories, rounds, evaluating the effects of medication, treatment, and nursing for systematic assessment.

Nurses, in particular, communicate interactively during the handover process, collect data fragments, and detect cues [27]. In this study, nurses reviewed electronic nursing records (ENRs) to understand the patient’s condition before handover, listened and asked questions to clarify patient data during the handover process, and directly observed and questioned the patient during rounds after handover. Pfrimmer et al. [27], who analyzed handover among intensive care unit nurses, defined nursing surveillance as “find meaning”, an effort to interpret and synthesize data, and described the process of finding meaning as “knowing the patient”, “sharing understanding and making decisions”, and “thinking ahead.” In conducting nursing surveillance, nurses used systematic assessment to identify necessary data to understand the big picture, while thinking about the patient’s image in their heads based on the data collected through a systematic assessment [27].

In this study, “pattern recognition” was similar to Halverson and Tilley’s [4] “pattern recognition”, which refers to nurses checking previous data, comparing current conditions, and applying experience to analyze changes in patient conditions. Henneman et al. [26] stated that monitoring and surveillance both involve data collection, but surveillance also includes data analysis. In addition, Halverson and Tilley [4] explained that nurses can anticipate problems by collecting and interpreting relevant data to identify patterns in patient conditions. Therefore, analyzing the data collected by nurses, identifying patterns, and predicting problems are essential for nurses to make clinical decisions about patient care [27,28], which were interpreted as cognitive factors of nursing surveillance.

Regarding “decision-making”, the study found that nurses often report to physicians when they detect changes or problems in a patient’s condition, and they participate in this decision-making by suggesting the necessary treatments or procedures to physicians. In general, a nurse’s clinical decision-making process includes cognitive processes directly related to nursing patients, such as creating a list of a patient’s problems or diagnoses and selecting appropriate interventions or treatments [29]. As a result, it includes both the tasks in which nurse-initiated interventions are the primary alternative, and tasks in which physician-initiated interventions are the primary alternative [30,31].

During the process of performing surveillance, nurses determine what data to collect and make decisions within the scope of their duties [2]. The list of 46 activities for surveillance intervention in the NIC [32] guides nursing activities based on their decision-making, including “selecting appropriate patient indicators for continuous monitoring based on the patient’s condition”, “determining trigger factors for patients who require immediate response”, “initiating or changing treatment plan using established protocols”, “recording type and amount of drainage and reporting significant changes to the physician”, “performing invasive hemodynamic monitoring, in collaboration with physicians if necessary”, and “monitoring intracranial pressure, in collaboration with physicians if necessary”, “seeking advice from physicians if patient data requires changes”, and “seeking advice from appropriate healthcare professionals for starting new treatments or changing existing ones.” In contrast, in this study, it was found that nurses mainly report to physicians and perform treatment or procedures according to physicians’ orders, but when there is a protocol established by the medical team, they perform urgent treatment first and report to the attending physician later. These results supported the need protocols that was developed by multidisciplinary collaboration for nurses to assess patients, anticipate problems, and deal with the problems appropriately [6,20]. In addition, to improve bedside nursing surveillance, Henneman et al. [26] proposed the development of a clinician decision support system to support nursing surveillance processes using evidence-based practice, including checklists to guide nurses in identifying the risk factors for complications and information technology (IT). Therefore, to ensure patient safety in acute care hospitals and support nurses’ decision-making and response, it is necessary to develop a decision support system, including surveillance checklists, and develop and implement protocols that allow nurses to exercise their judgment to ensure patient safety in emergencies through consultation with the healthcare team.

The desired outcome of nursing surveillance is to minimize adverse events for patient safety, and, in patient groups where nursing-surveillance-related nursing diagnoses were applied, more vital signs were measured, and in patient groups where more observations and monitoring were performed, the cardiac arrest recovery rate was higher [19,33]. In that study, nurses described the positive outcomes of surveillance as a “smoothly progressing gear”. Specifically, nurses reported that when surveillance is appropriately performed, it can prevent emergencies, allow for the rapid response to emergencies, help patient recovery, shorten hospitalization periods, prevent unnecessary treatment, increase nursing satisfaction, increase nurse confidence and satisfaction, and increase hospital trust. These results supported the findings of previous studies that suggest that if nursing surveillance is appropriately performed, complications and adverse events decrease, and patient and nursing satisfaction increase [4,20]. Additionally, the findings are similar to those of Kutney-Lee, Lake, and Aiken [34], who found that the quality of care and prevention of falls and nosocomial infections were higher in hospitals with higher levels of nurse education, experience, and expertise; adequate nurse staffing levels; and supportive work environments.

Meanwhile, critical thinking, clinical reasoning, intuition, and expertise are required for nurses to make decisions that correspond appropriately to subtle changes in a patient’s condition [5]. In this study, nurses perceived that to provide surveillance adequately, they needed specialized knowledge of the diseases and treatments of patients in their specialty, and at least 3 years of clinical experience. Nurses’ education and expertise were suggested as the antecedents of nursing surveillance [34]. In South Korea, where nursing education is unified into four years system, nurses perceived that the competence of new nurses with less than 1 year of experience after graduating from nursing school was insufficient to perform surveillance appropriately. Therefore, to ensure patient safety in acute care hospitals, it is necessary to develop and apply practical training and simulations to enhance the surveillance capabilities of newly hired nurses to identify the patient’s condition, anticipate problems, and respond appropriately. In addition, there is a need to develop ENR systems to support nursing surveillance for newly hired nurses [19,22,35].

In particular, in this study, nurses perceived that direct nursing time should be increased to allow nurses to frequently assess and identify patients to adequately perform surveillance. In South Korea, nurses are reported to be responsible for an average of 15.6 patients in acute hospitals, which is higher than in the U.S. (5 patients), Australia (5.3 patients), Japan (10 patients), and the United Kingdom (8.6 patients) [36]. It has been pointed out that the standards of Korean medical law do not reflect changes in the healthcare industry, such as an increase in the severity of patient conditions, an increase in nursing intensity, and an expansion of nursing roles. Thus, essentially, for nurses to provide adequate surveillance and sufficient direct nursing care to their patients, the number of patients each nurse is responsible for needs to be reduced.

The original ENR system was reported to have the following benefits: reduced chart retrieval time, verification of clinical tests, reduced transcription time, improved record keeping, streamlined reimbursement, improved communication among medical staff, and increased ability to monitor and surveil patient conditions [37,38]. However, regarding ENR systems used in most acute care hospitals in Korea, nurses reported that ENR systems do not help them provide personalized care for each patient [35]. In this study, nurses reported that the amount of paperwork and forms they had to fill out in the ENR system took their time from providing direct patient care. Time to providing direct nursing care is an important indicator that positively affects patient outcomes, and it is necessary to explore ways to increase direct care time, such as ensuring that nurses have time to perform surveillance appropriately, reasonably improving the recording in the ENR system, and using assistants for routine recording.

Based on the theoretical concept of nursing surveillance model, this study analyzed field data to identify the attributes and empirical cases of nursing surveillance as perceived by Korean nurses, and identify the factors promoting and hindering the nursing surveillance concept. However, this study has limitations in the generalizability of the results because the subjects were recruited by convenience sampling during the fieldwork phase, and academic journals only published in English and Korean were searched in the theoretical phase. Based on the conceptual analysis of nursing surveillance, it is recommended that future research should analyze nursing surveillance activities according to the attributes of nursing surveillance, empirically identify the antecedents and consequences of nursing surveillance, and develop the scales to measure nursing surveillance.

## 5. Conclusions

Based on the theoretical basis of the nursing surveillance concept, this study analyzed the surveillance experience of nurses working in acute care hospitals in South Korea. The attributes of nursing surveillance were identified as systematic assessment, pattern recognition, anticipation of problems, effective communication, decision-making, and performing nursing practice. This study has the significance of providing the conceptual framework of nursing surveillance for the development of the scales and suggests strategies to promote nursing surveillance in nursing organizations.

## Figures and Tables

**Table 1:** Nursing surveillance in the theoretical phase.

Authors (Year)	Antecedents (RN)	Antecedents (Work Environment)	Attributes	Facilitate NS	Consequences (Adequate NS)	Barriers to the Implementation of NS	Consequences (Inadequate NS)
Peet et al. (2022) [18]	Clinical experience, asserted nurses’ clinical knowledge, critical reflection, psychological safety, holistic facilitation	Learning culture, patient safety culture, safe, and effective ward culture	Purposeful and collaborative assessment, clinical reasoning, observing handovers, complexity, collaborating with staff, exploring alternative solutions	Collaborative relationship, person-centered relationships by both patients and staff, hourly mandated, protocolized, scripted bedside rounding, sense of harmony and teamwork, shared vision	Build safety cultures, effective safe teamwork, and nurses’ confidence	Communication hierarchies, time-pressured handover, adverse event data and workload systems, nurse leadership disengagement, rising conflict with doctors, constant negative audit feedback, time-consuming meeting, underestimating the complex environment	Clinical deterioration, frustrating team communication, and teamwork
Halverson and Scott (2022) [19]	Nurse certification, nurse education, nurse expertise	Organizational culture—hiring, staffing—retention, healthcare facility, and medical equipment, a culture of effective interdisciplinary communication, clinical ladder programs, support system	Systematic process, pattern recognition, the anticipation of the problem of instability, coordinated communication	Teamwork, strong clinical resources, standardized assessment, and intervention tools, coordinated communication (nurse and doctor), evidence-based handoff techniques and formal systems, good mix of nursing staff expertise	Patient safety, reduction in adverse events, nurse satisfaction, decreased nurse burnout	Abrupt communication, reporting fear of retaliation, nurse handoff of omitted and missed care, interruption and interfering nursing care	Adverse outcomes, errors, nurse burnout
Perry-Woodford and Marinova (2022) [20]	Understanding patients’ specific concerns, highly skilled nursing competency	Patient pathway	Quality of advice, good working relationships with patients	Protocol, patient feedback, and audit	Patient satisfaction, reducing patients’ problems improvement in patients’ perception of QoL, and confidence	Not feeling well-informed, no solutions to patients’ problems	Restriction on patients’ life
LeBlanc et al. (2022) [21]	Nurses’ knowledge, confidence, concerns	Competency-based education, training, simulation, creative learning strategies, nurse-to-patient ratios, adequate staffing, professional courtesy, respect, and trust	Detecting changes in condition early and providing appropriate urgent medical care and treatment, purposeful and ongoing acquisition, interpretation, and synthesis of patient data for clinical decision-making, nurse monitoring, swift and appropriate interventions	Standardized tool and handoff sheet, critical information regarding the patient’s current condition and treatment, rapid response teams	Keeping the patient safe and decreasing failure to rescue, maximizing optimal patient outcomes, professional courtesy, and trust, working in sync with each other	Lack of adherence to policies, procedures, and shortcuts with the delivery of care, and the inability to provide nursing surveillance	Medical errors
Christabel et al. (2022) [22]	Willing to participate in training and upgrading knowledge	Adequate designated workforce with explicitly identified duties, ample lines of communication, safe working environment	Effective prevention, detection, and control of the problem, maintaining close contact with the laboratory findings, cooperation	Staffing/less paperwork, standardized manner, user-friendly computer program, healthcare personnel-related professionalism, role boundaries, daily workflow and management, interdisciplinary collaboration, standards and protocols, and technological infrastructure	High-quality healthcare	Uncertain about the process, inactive role in the surveillance process, increased work demands and staff shortage, no computer programs available to support NS	
Stotts et al. (2020) [6]	Nurse education, experience, expertise, confidence, timely recognition and mitigation of clinical deterioration, collaboration	Staffing, standardized assessment, communication tools, availability of emergency services, team composition, opportunities for multidisciplinary care planning	Assessment, documentation, decision-making, intervening, communication, continuous process of acquisition, interpretation, recognition, and management of clinical deterioration and synthesis of physical, behavioral, and cognitive patient data to determine intervention and threats to health and safety during the course of nursing care	Availability of medical equipment, staffing, skill and team composition, interactions, standardized tools between people, and with technology	Increasing nursing confidence and assertion of concerns and escalation		Clinical deterioration, adverse events, high stakes, time-dependent work
Halverson et al. (2022) [23]			Purposeful, ongoing acquisition, interpretation, and synthesis of patient data for clinical decision-making, follow-up rounding, systematic process, pattern recognition, effective communication, and decision-making, physiological measurements associated with deterioration, continuous monitoring	EWS, consensus-approved assessment tools, vital sign-directed protocol, pre-MET UCR	Patient safety, detection of early deterioration signs		High incidence of pre-MET activation failure
Halverson and Scott (2022) [4]	Nurse education, expertise, staffing	Organizational culture	Systematic process, pattern recognition, anticipating problems, coordinated communication, decision-making	Assessment tool: EWS, NEWS, APACHE, checklists, follow-up rounding, coordinated handoff, pre-MET	Decreases complications, adverse outcomes, and nurse burnout, increased nurse satisfaction	Reactive care, uncoordinated care, siloed round	Adverse outcomes, medical errors, nurse burnout
Copanitsanou and Santy-Tomlinson (2021) [24]	Education, early recognition, and diagnosis of wound infection	Given feedback on their performance, hospital surveillance policies, effective diagnostic processes			Patient safety, effectiveness, and improvement of healthcare services	Scarcity of resources, lack of trained staff, microbiology support, and information technology support	Wasted energy and money
Allen et al. (2020) [25]	Knowledge and skill set, authority and training, expression of concern	Regulations, adjusting their workflow, and collaboration with each other	Direct and continuous observation and monitoring, recognize signs, accurately assess the probability	Changing their standards of practice to reflect what they know, believe, and value	Decreased duration of mechanical restraint episodes, quality improvement	Improper staffing, mandate	Increased risk and complications

Notes: RN, registered nurse; NS, nursing surveillance; EWS, early warning score; MET, medical emergency team; UCR, urgent review; NEWS, national early warning score; APACHE, Acute Physiology, and Chronic Health Evaluation.

**Table 2:** Characteristics of the participants (N = 10).

No.	Sex	Age	Education	Position	Total Career (Years)	Current Department	Current Department Career (Years)	Nursing Care Confidence
1	Female	27	BSN	General nurse	4.92	M	4.92	8
2	Female	30	BSN	General nurse	7.25	M	7.25	7
3	Female	37	MSN	General nurse	14.42	HRC	2.25	9
4	Female	38	BSN	Head nurse	15	M	15	8
5	Female	32	MSN	General nurse	9.67	ICU	8.08	7
6	Female	32	MSN	General nurse	8.33	M-S	8.33	9
7	Female	49	MSN	Charge nurse	28.83	S	1.25	10
8	Female	48	MSN	Charge nurse	24.83	IR	9	9
9	Female	33	BSN	Charge nurse	13	S	3	6
10	Female	30	BSN	General nurse	5.75	ICU	5.75	8

Notes: BNS, Bachelor of Science in Nursing; MSN, Master of Science in Nursing; M, medical part; HRC, Hospital Referral Center; ICU, intensive care unit; M-S, med-surgical part; S, surgical part; IR, injection room.

**Table 3:** Nursing surveillance in the fieldwork phase.

Antecedents (Situation)	Antecedents (RN)	Antecedents (Work Environment)	Attributes (Subcategories)	Participant Statements	Consequences (Adequate NS)	Consequences (Inadequate NS)
Handover, circumstances different from handover details, sudden change in patient condition after surgery or treatment, the occurrence of disease complications, urgent care, and emergencies	3–4 years of clinical experience, nursing proficiency, knowledge of disease and nursing, passion and will to actively solve problems, nursing professionalism, responsibility, rapport formation, intuition, interest, and empathy for patients, communication skills, careful observation, self-health and emotional control	Support of auxiliary personnel, patient severity, securing time for direct nursing care, staffing according to nursing proficiency, reducing the patient-to-nurse ratio, support and encouragement from colleagues, doctor–nurse cooperative relationship and sense of fellowship, sharing of treatment plans with doctors, practical training by the department, new nurse education and adaptation training	Systematic assessment	Carefully listening to handover details, direct confirmation of handover details (V/S, hemodynamic monitoring, consciousness, appearance, existing lines and drains, catheters, medication, order, lab, video, assessment through open questions to patients and guardians, disease characteristics focus assessment, treatment, treatment, postintervention effect assessment), acquisition of problematic cues, checking of alarms, past events, patient monitoring settings 24 h before handover and trend of patient condition change, continuous monitoring of the patient condition, planned rounds, checking the effectiveness of nursing intervention and treatment	The rapid and appropriate response to emergencies, patient safety, improvement of patient care quality, rapid recovery of patients and reduction in patient length of stay, patient’s cooperation and satisfaction, nurse’s job satisfaction and pride, increasing confidence in nursing performance, and hospital awareness and rapport with patients, doctors, and colleagues, decreasing nurse burnout and turnover, and frequency of unexpected emergencies, smooth progress as if a cogwheel was being engaged, reducing patient costs, preventing unnecessary treatment, passing on experiences to junior nurse	Deterioration of patient condition, death of a patient, an accusation of patient, guardian, doctor, and colleague, increase in customer’s complaints and patient medical costs, increasing nurse’s workload, working hours, fatigue, burnout, stress, and training costs, increase in medical lawsuits, financial loss, loss of trust with patients, medical staff, colleagues, and other departments, decreasing hospital satisfaction, increasing fear of patient care, guilt, excessive treatment
			Pattern recognition	Arranging handover details, organizing patient assessment data, integrating handover details and assessment data, interpreting the meaning of assessment data, identifying patterns by checking and comparing past and current assessment data, thinking about and finding factors that cause changes in patient conditions, recognizing patient problems to be solved during duty, treatment and nursing goals, recalling past patient experiences, evaluation of treatment and intervention effects		
			Anticipation of problem	Prediction of the patient’s problem situation through past and present data, including baseline data; prioritization of problems to be solved during duty; detection of problems and prediction of them by comprehensively judging the assessment data before the patient’s condition changes; disease progression, treatment effects, comparison with past patterns, predicting disease progression and doctor’s orders, preparing patients, and providing nursing care		
			Effective communication	Communicating with patients and guardians, communicating with handover nurses and duty nurses on the same shift, notifying doctors of perceived problems, patient condition, and changes after intervention		
			Decision-making	Discussion and proposal of the patient treatment plan, participation in patient treatment decision-making, the suggestion of consulting with other departments		
			Performing nursing practice	Carrying out nursing care according to orders or protocols, educating and preparing patients and guardians about predicted problems, symptom reduction, and problem-solving nursing, preparing for and carrying out predicted doctor’s orders, performing interventions that have to be carried out on time		

Notes: RN, registered nurse; NS, nursing surveillance; V/S, vital signs.

**Table 4:** Nursing surveillance in the final analysis phase.

Antecedents (Situation)	Antecedents (RN)	Antecedents (Work Environment)	Attributes		Consequences (Adequate NS)	Consequences (Inadequate NS)
-Handover -A sudden change in the patient’s condition -Urgent care and emergencies	-Clinical experience -Knowledge -Expertise -Professionalism -Responsibility -Communication skills -Self-health and emotional control	-Supporting resources -Securing time for direct nursing care -Proper nurse staffing -Cooperative relationship with staff -Education and adaptation program -Organizational culture -Clear standards and guidelines	Systematic assessment	-Purposeful and collaborative assessment -Acquisition of problematic cues -Continuous monitoring of the patient’s condition -Planned round -Checking the effectiveness of nursing intervention and treatment	-Patient safety -Improvement of patient care quality -Patient’s cooperation and satisfaction -Increasing nurse’s job satisfaction, confidence, hospital awareness, and rapport with patients, doctors, and colleagues, -Preventing unnecessary treatment -Passing on experiences to junior nurse	-Deterioration of the patient’s condition -Accusation of patient, guardian, doctor, and colleague -Increasing patient medical costs, nurse’s workload, burnout, stress, and training costs -Increase in medical lawsuits, financial loss, loss of trust
			Pattern recognition	-Organizing and synthesizing handover details and assessment data -Interpreting the meaning of assessment data -Identifying patterns by checking and comparing past and current assessment data -Recognizing patient problems to be solved during duty -Evaluating treatment and intervention effects		
			Anticipation of problems	-Prioritization of problems to be solved during duty -Detection of problems and prediction by comprehensively judging the assessment data before the patient’s condition change -Progress of the disease, and the treatment effect -Comparing with past patterns, predicting disease progression, and doctor’s order -Preparing for patients and nursing care		
			Effective communication	-Effective and coordinated communication with patients, guardians, handover nurses, duty nurses on the same shift, and other departments -Notifying doctors of perceived problems, patient conditions, and changes after the intervention		
			Decision- making	-Participation in the decision-making for patient treatment plans -Exploring alternative solutions		
			Performing nursing practice	-Performing nursing care according to orders, protocols, guidelines, and standards for clinical deterioration and problem-solving -Carrying out duty after predicting and preparing doctor’s orders -Performing interventions that have to be carried out on time		

Notes: RN, registered nurse; NS, nursing surveillance.

## Data Availability

Data are unavailable due to privacy or ethical restrictions.
